# Role of hippocampal p11 in the sustained antidepressant effect of ketamine in the chronic unpredictable mild stress model

**DOI:** 10.1038/tp.2016.21

**Published:** 2016-02-23

**Authors:** H-L Sun, Z-Q Zhou, G-F Zhang, C Yang, X-M Wang, J-C Shen, K Hashimoto, J-J Yang

**Affiliations:** 1 Department of Anesthesiology, Jinling Hospital, School of Medicine, Nanjing University, Nanjing, China; 2 Division of Clinical Neuroscience, Chiba University Center for Forensic Mental Health, Chiba, Japan

## Abstract

Although ketamine shows a rapid and sustained antidepressant effect, the precise mechanisms underlying its effect are unknown. Recent studies indicate a key role of p11 (also known as S100A10) in depression-like behavior in rodents. The present study aimed to investigate the role of p11 in the antidepressant-like action of ketamine in chronic unpredictable mild stress (CUMS) rat model. The open-field test, forced swimming test and sucrose preference test were performed after administration of ketamine (10 mg kg^−1^) or a combination of ketamine and ANA-12 (a tropomyosin-related kinase B (TrkB) antagonist; 0.5 mg kg^−1^). The lentivirus vector for p11 was constructed to knock down the hippocampal expression of p11. In the CUMS rats, ketamine showed a rapid (0.5 h) and sustained (72 h) antidepressant effect, and its effect was significantly blocked by co-administration of ANA-12. Furthermore, ketamine significantly increased the reduced expression of brain-derived neurotrophic factor (BDNF) in the hippocampus of CUMS rats, whereas ketamine did not affect the expression of p11 in CUMS rats 0.5 h after administration. In addition, ketamine significantly increased the reduced ratio of p-TrkB/TrkB in the hippocampus by CUMS rats, and its effect was also blocked by ANA-12. Moreover, the reduced expression of BDNF and p11 in the hippocampus of CUMS rats was significantly recovered to control levels 72 h after ketamine administration. Interestingly, knockdown of hippocampal p11 caused increased immobility time and decreased sucrose preference, which were not improved by ketamine administration. These results suggest that p11 in the hippocampus may have a key role in the sustained antidepressant effect of ketamine in the CUMS model of depression.

## Introduction

Depression is one of the most common psychiatric disorders affecting nearly 20% of the population worldwide, and more than half of the suicides are accompanied by depression.^1, 2, 3, 4, 5^ Antidepressants such as selective serotonin reuptake inhibitors (5-hydroxytryptamine, 5-HT) and noradrenaline reuptake inhibitors are the clinically prescribed drugs for the treatment of depression. However, it takes several weeks for these drugs to exert the antidepressant effects; moreover their remission rates are only approximately 40%.^4, 6^ Therefore, more studies are urgently needed to find a new, effective approach and to examine the pathophysiology of depression.

A number of studies have shown that a single subanesthetic dose of ketamine, a noncompetitive *N*-methyl-d-aspartate antagonist, produces rapid and sustained antidepressant effects in animal models^7, 8, 9^ and in treatment-resistant patients with major depressive disorder and bipolar disorder.^10, 11, 12, 13^ Several molecular mechanisms, neural circuits and signal transduction pathways have been involved in the mechanism of ketamine's antidepressant effect; however, the precise mechanisms underlying its antidepressant effect remain largely to be determined.^14, 15, 16, 17, 18, 19^

The protein p11 (also known as S100A10), a member of the S100 EF-hand protein family, is widely expressed in several brain regions that are implicated in the pathophysiology of depression, including the hippocampus and frontal cortex.^20, 21, 22^ Accumulating evidence suggests a key role of p11 in the pathophysiology of depression. The levels of mRNA and protein of p11 are downregulated in the hippocampus of rodents with depression-like phenotype and the peripheral blood mononuclear cells of depressed patients.^23, 24^ Furthermore, decreased p11 mRNA levels are also observed in the hippocampus of suicide victims.^25^ Interestingly, the overexpression of p11 can rescue the depression-like phenotype in p11 knockout mice, and selective serotonin reuptake inhibitors or tricyclic antidepressants promote the expression of p11 in the frontal cortex and hippocampus of rodents.^23, 26^

Clinical and preclinical studies report that ketamine can increase brain-derived neurotrophic factor (BDNF), and that BDNF and its receptor tropomyosin-related kinase B (TrkB) may have a role in the antidepressant-like activity of ketamine.^9, 27, 28, 29^ Interestingly, it is reported that BDNF can regulate the expression of p11 *in vivo* and *in vitro*.^30^ However, it remains unknown whether p11 participates in the antidepressant-like activity of ketamine. Therefore, the purpose of this study was to investigate the role of p11 in the rapid and sustained antidepressant-like activity of ketamine in chronic unpredicted mild stress (CUMS) rats.

## Materials and methods

### Animals

Adult male Sprague Dawley rats (250–300 g) purchased from comparative medicine of Jinling Hospital were housed at 21 °C and maintained on 12-h light/dark cycle (light on at 0700). Food and water was obtained *ad libitum*. This study was approved by the Ethics Committee of Jinling Hospital, Nanjing, China, and performed in accordance with the Guide for the Care and Use of Laboratory Animals from the National Institutes of Health, USA.

### Design and drug interventions

A total of 96 rats were divided into seven groups: control group (*n=*24), CUMS+saline group (*n=*16), CUMS+ketamine group (*n=*16), CUMS+ketamine+ANA-12 (a selective TrkB antagonist)^31^ group (*n=*16), LV-eGFP (lentivirus with enhanced green fluorescent protein) group (*n=*8), LV-p11-eGFP+saline group (*n=*8) and LV-p11-eGFP+ketamine (*n=*8).

Ketamine hydrochloride (Gutian Pharmaceutical Company, Fujian, China) at 10 mg kg^−1^ was intraperitoneally administered 0.5 h or 72 h before the behavioral tests. ANA-12 (0.5 mg kg^−1^, Sigma Chemical, St Louis, MO, USA) was intraperitoneally administered with ketamine (10 mg kg^−1^).

### CUMS protocol

The depression model was set up by the CUMS as described previously with a slight modification.^32^ The rats in the CUMS groups were exposed to 10 different stressors for 20 days (two stressors per day), namely, 24 h food deprivation, 24 h water deprivation, 24 h 45° tilted cages, damp bedding, lights on overnight, lights off daytime, 5-min rotation on a shaker, placement in a 4 °C environment, isolation and crowding. At day 21, the rats were forced to swim for 15 min in a cylindrical tank (diameter 30 cm, height 75 cm) filled with 22 °C water (depth 40 cm).

### Lentivirus production, screening and stereotaxic injection

To silence p11 in the hippocampus, three lentiviruses (LVs) targeting different sequences of p11 were constructed (Gene Chem, Shanghai, China). The p11 LV-A (5′-CCTGAGAGTGCTCATGGAA-3′), LV-B (5′-TCCCAAATGGAGCATGCCA-3′) and LV-C (5′-GTACACATGAAGCAGAAGA-3′) were screened by western blotting to determine which LV can maximally silence the expression of p11 in neural culture cells.

Primary hippocampal neurons were isolated from 18-day timed pregnant Sprague Dawley rats, as previously described.^33^ Neurons were cultured for 7 days *in vitro* in neurobasal B27 (1:50 dilution; Invitrogen, Shanghai, China) supplemented medium (Gibco, Invitrogen), and then the LV-p11-eGFP with three different sequences and LV-eGFP were applied for 5 days *in vitro*. At 12 days *in vitro*, the cultured hippocampal neurons were dissociated and western blotting was used to test the expression of p11.

The screened LV-p11-eGFP or LV-eGFP was administered into the dentate gyrus (DG) of the hippocampus by stereotaxic instrument at 10 days before drug treatment. Briefly, rats were anesthetized with sodium pentobarbital (60 mg kg^−1^, intraperitoneally) and fixed on the stereotaxic frame. Then, the LV-p11-eGFP or LV-eGFP was injected into the DG region of the ventral hippocampus (anteroposterior, −4.5 mm; mediolateral, ±3.0 mm; dorsoventral, −4.0 mm) at a rate of 0.2 μl min^−1^. After injection, the needle was kept in the brain for another 3 min. After surgery, the animals were placed under a heating lamp until awakening and further monitored daily.

### Histology

Thirteen days after LV-eGFP or LV-B injection, the rats were killed and the brains were fixed by transcardial perfusion with 0.1 m PBS for 10 min after the rats were deeply anesthetized with sodium pentobarbital (60 mg kg^−1^, intraperitoneally). Brains were removed and kept in 25 ml 4% PFA for 1 h and then were washed in 0.1 m PBS and immersed in 15% and subsequently 30% sucrose solution for 2 days. The brains were blotted dry and snap-frozen for 10 s in isopentane on dry ice and stored at −80 °C until sectioning. Serial coronal 20-μm-thick sections were obtained using a cryostat (Leica CM 1900, Leica, Wetzlar, Germany). All the brain sections containing the hippocampus were collected and thaw-mounted on Super Frost microscope slides. The slides were then stained with DAPI (4',6-diamidino-2-phenylindole) and confocal images were acquired using a confocal microscope (Leica, TCS SP2).

### Behavioral tests

The behavioral tests were conducted at 0.5 h or 72 h after drug administration or 13 days after LV-p11-eGFP injection.

#### Open-field test

Locomotor activities of the rats were measured with an open-field apparatus (75 × 75 × 40 cm). The rat was placed individually into the center of the field, and the total distance during 20 min was recorded by a camera.

#### Forced swimming test

The depressive behavior of the rats was evaluated by the forced swimming test (FST). The rats were placed in a cylindrical tank, as described in the CUMS protocol, for 6 min. The immobility time during the final 5 min was recorded by an observer who was blind to the treatment group. The immobility time was defined as the time during which the rat stood still without struggling or used only minimal movements to keep the head above water. The water was changed after each test.

#### Sucrose preference test

The anhedonia symptom of depression is reflected by the sucrose preference test (SPT). The rats were habituated to a 1% sucrose solution for 24 h after ketamine or saline administration. On the second day, rats were given access to only water. The animals were then allowed the 1% sucrose solution or the same volume of water. The locations of sucrose and water were exchanged after 12 h to avoid the place preference. Sucrose preference was defined as the percentage of sucrose solution consumed of the total fluid volume during 24 h.

### Western blotting

Western blotting was performed to test the expressions of p11, BDNF, proBDNF, TrkB and p-TrkB in the hippocampus of rat or cultured neurons. After the behavioral tests, the rats were anesthetized with sodium pentobarbital (60 mg kg^−1^), and the hippocampus was removed and homogenized on ice. The hippocampal neural cells were dissociated by cell lysis buffer. The BCA assay was used to determine the concentration of proteins. All the normalized samples were separated by 12 or 15% sodium dodecyl sulfate polyacrylamide gel electrophoresis, transferred onto polyvinylidene fluoride membranes, blocked by 3% bovine serum albumin and incubated with primary antibodies overnight at 4 °C, including p11 (1:500, Abcam, Cambridge, UK), BDNF (1:1000, Abcam), proBDNF (1:800, Abcam), TrkB (1:500, Abcam), p-TrkB (1:500, Abcam) and tubulin (1:1000, Abcam). The polyvinylidene fluoride membranes were washed with Tris-buffered saline plus Tween 20 and then incubated with second antibodies for 1 h at room temperature (rabbit or mouse anti-goat 1:8000). ImageJ software was used to calculate the gray value of immune reactivity.

### Statistical analysis

Data are expressed as mean±s.e.m. and were analyzed by Statistical Package for Social Sciences (SPSS version 17.0, SPSS IBM, Chicago, IL, USA). Statistical significance among groups was assessed by one-way analysis of variance (ANOVA), followed by *post hoc* Bonferroni tests. *P*<0.05 was considered statistically significant.

## Results

### Levels of hippocampal p11, BDNF, proBDNF, TrkB and p-TrkB in the rapid antidepressant-like activity of ketamine

**Figure 1:**
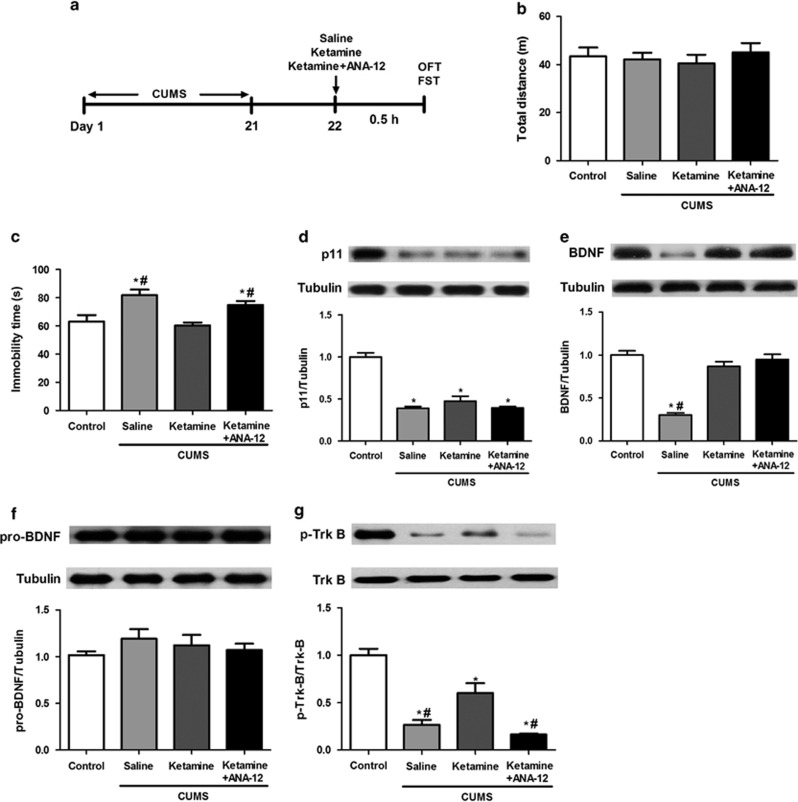
Behavioral tests and western blotting of p11, BDNF, proBDNF, and p-TrkB/TrkB in the hippocampus 0.5 h after ketamine (or ketamine and ANA-12) administration in CUMS rats. (**a**) The schedule of CUMS model and behavioral tests. CUMS was performed from day 1 to day 21. On day 22, saline (10 ml kg^−1^), ketamine (10 mg kg^−1^) or ketamine (10 mg kg^−1^) and ANA-12 (0.5 mg kg^−1^) were administered intraperitoneally into CUMS rats. Behavioral tests such as open-field test (OFT) and forced swimming test (FST) were performed 0.5 h after administration. (**b**) The total distance traveled by the rats in the OFT. (**c**) The immobility time of the rats in the FST. (**d**) The expression of p11 in the hippocampus of the four groups. (**e**) The expression of BDNF in the hippocampus of the four groups. (**f**) The expression of proBDNF in the hippocampus of the four groups. (**g**) The ratio of p-TrkB to total TrkB in the hippocampus of the four groups. *P<0.05, compared with the control group. ^#^*P*<0.05, compared with the ketamine-treated group of CUMS rats. BDNF, brain-derived neurotrophic factor; CUMS, chronic unpredictable mild stress; TrkB, tropomyosin-related kinase B.

To test the rapid antidepressant-like activity of ketamine, open-field test and FST were performed at 0.5 h after ketamine or saline administration (Figure 1a). No significant difference (F(3,28)=0.297, *P*=0.82) was found in the total distance among the four groups (Figure 1b). One-way ANOVA of FST data revealed significant differences among the four groups (F(3,28)=7.921, *P*<0.01). In the FST, the immobility time of the saline-treated group of CUMS rats was significantly (*P*<0.01) higher than that of the control group (Figure 1c). The immobility time of the ketamine-treated group of CUMS rats was significantly (*P*<0.01) lower than that of the saline-treated group of CUMS rats (Figure 1c). Furthermore, co-administration of ANA-12 significantly (*P*<0.05) blocked the antidepressant effect of ketamine in CUMS rats (Figure 1c).

One-way ANOVA of p11 data revealed significant differences among the four groups (F(3,8)=50.673, *P*<0.001). Levels of p11 in the hippocampus of CUMS rats were significantly (*P*<0.001) lower than those of the control rats (Figure 1d). Ketamine or a combination of ketamine and ANA-12 did not alter the reduced levels of p11 in the hippocampus of CUMS rats (Figure 1d).

One-way ANOVA of BDNF data revealed significant differences (F(3,8)=43.574, *P*<0.001) among the four groups. Levels of BDNF in the hippocampus of CUMS rats were significantly (*P*<0.001) lower than those of the control rats (Figure 1e). Ketamine or a combination of ketamine and ANA-12 significantly (*P*<0.01) increased the levels of BDNF in the hippocampus to control levels (Figure 1e).

One-way ANOVA of proBDNF data revealed no significant difference among the four groups (F(3,28)=0.752, *P*=0.55) (Figure 1f). The ratio of p-TrkB to total TrkB data revealed significant difference among the four groups (F(3,8)=31.803, *P*<0.01). The ratio of p-TrkB to total TrkB was significantly (*P*<0.001) lower in the CUMS rats than in the controls (Figure 1g). Ketamine significantly (*P*<0.05) increased the ratio of p-TrkB to total TrkB in the hippocampus of CUMS rats, which could be blocked by the co-administration of ANA-12 (Figure 1g).

### Levels of hippocampal p11, BDNF, proBDNF, TrkB and p-TrkB in the sustained antidepressant-like activity of ketamine

**Figure 2:**
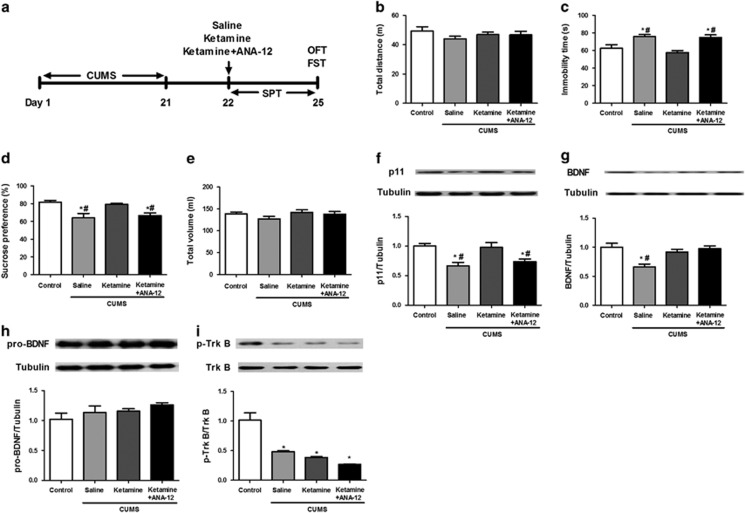
Behavioral tests and western blotting of p11, BDNF, proBDNF, and p-TrkB/TrkB in the hippocampus 72 h after ketamine (or ketamine and ANA-12) administration in CUMS rats. (**a**) The schedule of CUMS model and behavioral tests. CUMS was performed from days 1 to days 21. At day 22, saline (10 ml kg^−1^), ketamine (10 mg kg^−1^) or ketamine (10 mg kg^−1^) and ANA-12 (0.5 mg kg^−1^) were administered intraperitoneally into CUMS rats. Behavioral tests were performed 72 h after administration. (**b**) The total distance traveled by the rats in the OFT. (**c**) The immobility time of the rats in the FST. (**d**) The percent of sucrose preference of the rats in the SPT. (**e**) The total volume consumed by the rats in the SPT. (**f**) The expression of p11 in the hippocampus of the four groups. (**g**) The expression of BDNF in the hippocampus of the four groups. (**h**) The expression of proBDNF in the hippocampus of the four groups. (**i**) The ratio of p-TrkB to total TrkB in the hippocampus of the four groups. *P<0.05, compared with the control group. ^#^*P*<0.05, compared with the ketamine-treated group of CUMS rats. BDNF, brain-derived neurotrophic factor; CUMS, chronic unpredictable mild stress; FST, forced swimming test; OFT, open-field test; SPT, sucrose preference test; TrkB, tropomyosin-related kinase B.

To test the sustained antidepressant-like activity of ketamine, open-field test and FST were performed at 72 h and SPT during a 3-day period after administration of saline, ketamine or a combination of ketamine and ANA-12 (Figure 2a). The total distance among the groups had no significant difference (F(3,28)=0.980, *P*=0.42; Figure 2b). One-way ANOVA of FST data revealed significant differences among the four groups (F(3,28)=9.293, *P*<0.001). The immobility time of the saline-treated group of CUMS rats was significantly (*P*<0.05) higher than that of the control group (Figure 2c). The immobility time of the ketamine-treated group of CUMS rats was significantly (*P*<0.01) lower than that of the saline-treated group of CUMS rats (Figure 2c). Furthermore, co-administration of ANA-12 significantly (*P*<0.01) blocked the antidepressant effect of ketamine in CUMS rats (Figure 2c). One-way ANOVA of SPT data revealed significant differences among the four groups (F(3,28)=8.447, *P*<0.01). The sucrose preference of the saline-treated group of CUMS rats was significantly (*P*<0.01) lower than that of the control rats. The sucrose preference of the ketamine-treated group of CUMS rats was significantly (*P*<0.05) higher than that of the saline-treated group of CUMS rats (Figure 2d). Furthermore, co-administration of ANA-12 significantly (*P*<0.05) blocked the anti-anhedonia effect of ketamine in CUMS rats (Figure 2d). In contrast, in the SPT, there was no significant difference in the total volume consumed among the groups (F(3,28)=1.405, *P*=0.27; Figure 2e).

One-way ANOVA of p11 data revealed significant differences among the four groups (F(3,8)=7.992, *P*<0.01). Levels of p11 in the hippocampus of CUMS rats were significantly lower than those of the control rats (Figure 2f). Ketamine significantly attenuated the reduced levels of p11 in the hippocampus of CUMS rats (Figure 2f). Interestingly, co-administration of ANA-12 significantly blocked the effect of ketamine on p11 expression in the hippocampus (Figure 2f).

One-way ANOVA of BDNF data revealed significant differences among the four groups (F(3,8)=8.875, *P*<0.01). Levels of BDNF in the hippocampus of CUMS rats were significantly (*P*<0.05) lower than those of the control rats (Figure 2g). Ketamine or a combination of ketamine and ANA-12 significantly (*P*<0.05) increased levels of BDNF in the hippocampus of CUMS rats compared with the saline-treated group of CUMS rats (Figure 2g).

One-way ANOVA of proBDNF data revealed no significant difference (F(3,8)=1.605, *P*=0.263) among the four groups (Figure 2h). The ratio of p-TrkB to total TrkB data revealed significant difference (F(3,8)=27.328, *P*<0.001) among the four groups. The ratio of p-TrkB to total TrkB was significantly (*P*<0.01) lower in the CUMS rats than in the controls (*P*<0.001; Figure 2i). No significant difference was detected in the ratio of p-TrkB to total TrkB of the CUMS rats after ketamine or a combination of ketamine and ANA-12 administration (Figure 2i).

### The effects of knockdown of p11 in the sustained antidepressant-like activity of ketamine

**Figure 3:**
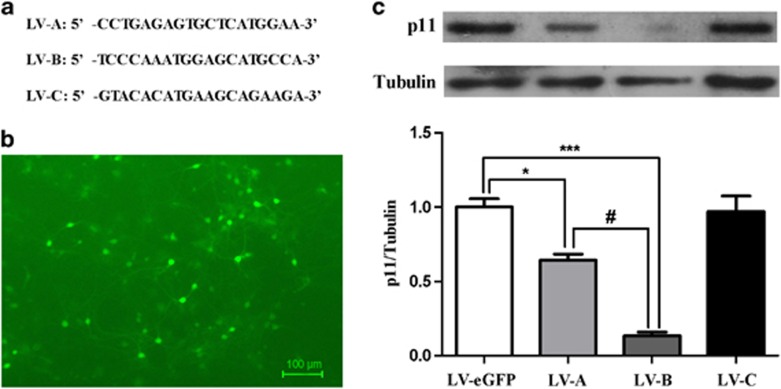
The efficiency of three different sequences LV-p11-eGFP. (**a**) The three different sequences of LV-p11-eGFP. (**b**) The primary hippocampal neurons were infected by LV-p11-eGFP *in vitro*. (**c**) The expression of p11 in the primary neuron cultures. *P<0.05, ***P<0.001, compared with the LV-eGFP group. ^#^*P*< 0.05, compared with the LV-A group. eGFP, enhanced GFP; GFP, green fluorescent protein; LV, lentivirus.

Three different sequences of LV-p11-eGFP were built to silence p11, which were screened by primary neuronal culture (Figures 3a and b). Both LV-A and LV-B, but not LV-C, decreased the expression of p11 in the cultured hippocampal cells (Figure 3c). One-way ANOVA of p11 data revealed significant differences among the four groups (F(3,8)=39.677, *P*<0.001). The LV-A reduced approximately 32.8% expression of p11, and the LV-B was approximately 86.5% (Figure 3c). Therefore, LV-B was selected to knock down the hippocampal p11 in the rats.

**Figure 4:**
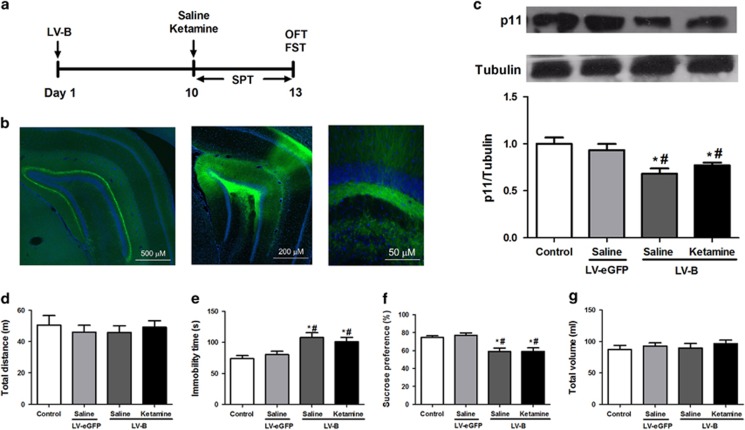
Effect of ketamine on depression-like behavior in rats after knockdown of hippocampal p11. (**a**) The schedule of hippocampal p11 knockdown and behavioral tests. LV-p11-eGFP or LV-B was injected into DG region of rat hippocampus. Ten days after injection, saline (10 ml kg^−1^) or ketamine (10 mg kg^−1^) was administered intraperitoneally into the rats. (**b**) Appearance of eGFP+ cells in ventral hippocampus DG post injection of the LV-B. (**c**) On day 13, the expression of hippocampal p11 was measured after behavioral tests. (**d**) The total distance traveled by the rats in the OFT. (**e**) The immobility time of the rats in the FST. (**f**) The percent of sucrose preference of the rats in the SPT. (**g**) The total volume consumed by the rats in the SPT. *P<0.05, compared with the control group. ^#^*P*<0.05, compared with the LV-eGFP group. eGFP, enhanced GFP; FST, forced swimming test; GFP, green fluorescent protein; LV, lentivirus; OFT, open-field test; SPT, sucrose preference test.

To knock down the hippocampal expression of p11 *in vivo*, the LV-B was bilaterally injected into the DG zone (Figure 4a). The hippocampal injection of LV-B was able to infect the surrounding neuronal cells during the 13-day interval (Figure 4b). The low magnification displayed that the DG region was effectively infected as exemplified by the eGFP^+^ cells across the entire region, particularly the mossy cell axons. At medium magnification, the heavily infected DG and CA1 regions were evident, and at high magnification, there were neurons and the axons of cells from the DG (the mossy fibers), which transmit information to other cells in the dentate and to area CA3. One-way ANOVA of p11 data revealed significant differences among the four groups (F(3,8)=10.710, *P*<0.01). Compared with the LV-eGFP group, the expression of hippocampal p11 protein was significantly (*P*<0.05) decreased in the saline-treated and ketamine-treated groups of LV-B-injected rats on day 13 after LV-p11-eGFP injection (Figure 4c). No significant difference was found in the p11 levels among the saline-treated group and the ketamine-treated group (Figure 4c).

To test the alterations of sustained antidepressant-like effects of ketamine after knockdowning, the hippocampal expression of p11 *in vivo*, open-field test and FST were performed at 72 h and SPT during a 3-day period after the administration of saline or ketamine (Figure 4a). There was no significant difference (F(3,28)=0.239, *P*=0.86) among groups in the total distance of the open-field test (Figure 4d). One-way ANOVA of FST data revealed significant differences among the four groups (F(3,28)=6.682, *P*<0.01). The immobility time of saline-treated and ketamine-treated rats within the LV-B group was significantly (*P*<0.05) higher than that of the control and LV-eGFP groups (Figure 4e). One-way ANOVA of SPT data revealed significant differences among the four groups (F(3,28)=9.271, *P*<0.001). The sucrose preference of the saline-treated and ketamine-treated rats within the LV-B group was significantly (*P*<0.05) lower than that of the control and LV-eGFP groups (Figure 4f). In the SPT, the total volume consumed among the four groups was not significantly different (F(3,28)=0.424, *P*=0.73; Figure 4g).

## Discussion

The major findings of the present study are as follows. First, ketamine produced a rapid and sustained antidepressant-like effect in CUMS rats, and the TrkB antagonist ANA-12 significantly blocked the antidepressant effect of ketamine. Second, hippocampal BDNF levels of CUMS rats were significantly lower than those of the control rats, and a single administration of ketamine to CUMS rats could increase hippocampal BDNF to control levels. Third, hippocampal p11 levels of CUMS rats were significantly lower than those of control rats. Ketamine did not alter the expression of p11 in the hippocampus of CUMS rats when measured 0.5 h after administration. However, the expression of p11 in the hippocampus of CUMS rats was recovered to control levels 72 h after ketamine administration. This effect was blocked by co-treatment with ANA-12. Fourth, the expressions of proBDNF in the hippocampus from CUMS rats treated with saline, ketamine or a combination of ketamine and ANA-12 had no significant differences compared with the control rats. Fifth, the reduced ratio of p-TrkB to total TrkB in the CUMS rats was improved 0.5 h after ketamine administration, and this effect was blocked by co-treatment with ANA-12. Sixth, the knockdown of p11 in the hippocampus induced depression-like behavior in rats, which was not improved by the administration of ketamine. Altogether, these findings suggest that BDNF–TrkB signaling and p11 in the hippocampus have key roles in the sustained antidepressant effect of ketamine.

Consistent with previous reports, this study found a rapid and sustained antidepressant effect of ketamine in the CUMS model of depression.^7, 8, 9^ Currently, the precise mechanisms underlying the effect of ketamine are still unclear. By blocking the *N*-methyl-d-aspartate receptors, ketamine-induced glutamate can activate AMPA receptors resulting in the activation of intracellular cascades, including mammalian target of rapamycin, cAMP response element-binding protein and postsynaptic density protein (PSD-95).^7, 9, 15^ Multiple studies have suggested a key role of BDNF–TrkB signaling in the pathophysiology of depression and in the therapeutic mechanism of antidepressants.^2, 34, 35, 36^ The hippocampal expression of BDNF is decreased in rodent models of depression, and chronic treatment with antidepressants increases BDNF expression in the hippocampus.^2, 34, 37, 38^ BDNF is synthesized by the proteolytic cleavage of proBDNF that catalyzed by the plasmin.^39^ However, it is reported that BDNF and proBDNF show opposite effects on physiological function. ProBDNF preferentially binds to p75 neurotrophin receptors, triggering anti-plasticity and pro-apoptotic actions, while BDNF has high affinity to TrkB receptors, which promotes neuronal cell survival, modulates synaptic plasticity and facilitates hippocampal neurogenesis, all of which are related to the cellular actions of antidepressants.^9, 39, 40^ In this study, we found that the hippocampal levels of proBDNF and TrkB had no significant difference among groups, while the levels of BDNF and p-TrkB/TrkB ratio in the hippocampus of CUMS rats were increased at 0.5 h after ketamine administration, indicating that the rapid antidepressant effect of ketamine may be mediated by the stimulation of BDNF–TrkB signaling in the hippocampus. Meanwhile, we also found that hippocampal BDNF levels of CUMS rats were increased at 72 h after ketamine administration. These results suggest that the BDNF–TrkB signaling in the hippocampus has a role in the rapid and sustained antidepressant-like activity of ketamine.

It is reported that BDNF could increase the expression of p11 in primary hippocampal culture, and the increase of p11 protein by 5-HT was attenuated in primary hippocampal culture from BDNF knockout mice.^30^ Furthermore, the expression of mRNA and protein of p11 in the brain of BDNF knockout mice was significantly lower than that of the wild-type mice.^30^ Moreover, the p11 knockout mice showed depression-like behavior in the tail suspension test and FST, and BDNF did not show an antidepressant effect in these mice.^30^ These findings suggest a key role of p11 in the antidepressant effect of BDNF. In this study, we found that the expression of p11 in the hippocampus of CUMS rats was increased at 72 h after a single administration of ketamine; which could be blocked by ANA-12. In contrast, we found that knockdown of p11 in the rat hippocampus produced depression-like behavior and that ketamine did not show antidepressant effects in the rats with knockdown of hippocampal p11. Thus, it is likely that CUMS causes the reduction of BDNF in the hippocampus, followed decreased p11 expression, resulting in depression-like behavior in rats. Furthermore, BDNF–TrkB signaling and p11 in the hippocampus have key roles in the sustained antidepressant effect of ketamine.

Unfortunately, we found no significant alteration of hippocampal p11 at 0.5 h after ketamine administration, suggesting that p11 may not have a role in the rapid antidepressant-like activity of ketamine. Warner-Schmidt *et al.*^30^ found that p11 protein was shown to increase at 3 h after BDNF stimulation in primary neural cultures. Therefore, it is unlikely that 0.5 h was sufficient time for the p11 protein to increase after ketamine administration. Meanwhile, Gigliucci *et al.*^41^ reported that the reduction of the immobility time of FST by ketamine was blocked by 5-HT depletion when ketamine was administered 24 h, but not 1 h, before FST. These results suggest the role of 5-HT in the sustained antidepressant effect of ketamine. 5-HT and its receptors were also involved in the expression of BDNF by antidepressants.^42, 43^ Interestingly, p11 also interacts with 5-HT receptors, including 5-HT_1B_, 5-HT_1D_ and 5-HT_4_ receptors.^22^ Therefore, the relationship between 5-HT receptors and BDNF–TrkB signaling may be involved in the role of p11 in the sustained antidepressant-like activity of ketamine. However, more detailed studies are needed to confirm this hypothesis.

How p11 mediates the therapeutic activity of antidepressants is still unknown. It has been reported that p11 facilitates surface expression of the 5-HT_1B_ and 5-HT_4_ receptors, modulates several cell process and interacts with a number of ion channels and G-protein-coupled receptors.^20, 23, 44^ Winterer *et al.*^45^ demonstrated that 5-HT via 5-HT_1B_ receptors reduces the feedback inhibition of interneurons, resulting in an increase in the excitation of CA1 pyramidal cells within the rat hippocampus. However, previous studies have also shown that 5-HT_4_ receptors in the hippocampus are more relevant to the antidepressant-like activity of p11 than 5-HT_1B_ receptors.^46^ This outcome is due to a higher level of 5-HT_4_ receptors in the hippocampus and the inhibited synaptic glutamate currents by activation of the presynaptic 5-HT_1B_ receptor.^47, 48^ Therefore, it should be noted that 5-HT_4_ receptors are also identified as the fast-acting antidepressant target.^49^

The subventricular zone and subgranular zone of the DG are two primary areas of adult neurogenesis.^50^ Ketamine produces antidepressant-like activity by increasing neurogenesis within the hippocampus, which is highly sensitive to chronic stress.^51^ A previous research study illustrated that the neurogenic effects of fluoxetine were attenuated in p11 knockout mice, suggesting that p11 might mediate the neurogenesis effects of antidepressants.^52^ Therefore, p11 might also mediate the antidepressant-like activity of ketamine with respect to neurogenesis. However, recent studies have reported that the open-field test and FST were neurogenesis-independent behavioral tests.^53^ Further studies to investigate the neurogenesis-dependent behaviors in the p11-mediated antidepressant-like activity of ketamine are interesting.

Finally, this study has a limitation that need to be mentioned. It is also known that prefrontal cortex and nucleus accumbens are involved in the depression-like phenotype.^15, 54, 55, 56^ Further studies will be needed to study the role of p11 in these two brain regions for the antidepressant action of ketamine.

In conclusion, the present study suggests that BDNF–TrkB signaling and p11 in the hippocampus have key roles in the sustained antidepressant-like activity of ketamine. Therefore, it is likely that p11 might be a new target for the development of ketamine-like antidepressants.
